# Adenoid Cystic Carcinoma of the Bartholin Gland: A Case Report

**DOI:** 10.7759/cureus.103953

**Published:** 2026-02-20

**Authors:** Marco F Bombon, Carlos J Alarcon, Jose L Reyes, Jenniffer S Plaza, Gema G Plaza

**Affiliations:** 1 Surgical Oncology, Sociedad de Lucha Contra el Cáncer (SOLCA) Matriz, Guayaquil, ECU; 2 Postgraduate Surgery, Universidad de Especialidades Espíritu Santo (UEES), Guayaquil, ECU; 3 Surgical Oncology and Gynecology, Sociedad de Lucha Contra el Cáncer (SOLCA) Matriz, Guayaquil, ECU; 4 Surgical Oncology, Hospital Juarez, Mexico, MEX

**Keywords:** adenoid cystic carcinoma, bartholin's gland, surgery, vulvar carcinoma, vulvar neoplasia, vulvectomy

## Abstract

Carcinoma of the Bartholin gland is a rare malignant tumor of the female genital tract, subcategorized within primary carcinomas of the vulva. The histopathological variant adenoid cystic carcinoma is extremely rare. There is no specific, consensus treatment for this pathology, but the gold standard is surgical removal.

We present the case of a 30-year-old female patient with a history of Barrett's esophagus, who underwent specific treatment and presented with a lesion in the right vulvar region that had been causing moderate pain and localized itching for several months. She underwent surgery: right hemivulvectomy plus inguinal lymphadenectomy with a histopathological result of adenoid cystic carcinoma of the Bartholin's gland.

Adenoid cystic carcinoma of the Bartholin's gland is a rare pathology with low incidence and unknown etiology. Diagnosis requires a combination of clinical, imaging, and histopathological aspects, and its treatment is currently controversial and nonspecific.

## Introduction

Bartholin's gland carcinoma is an extremely rare pathology of the female genital tract [1], mainly observed in postmenopausal women [2] between the ages of 50 and 67 [3]. Its etiology is not well defined [4]. These tumors have nonspecific clinical characteristics, and their differential diagnosis is complex, as they can be confused with other vulvar lesions [4]. However, when symptoms do occur, they are related to infiltration of the perineural area and manifest as pelvic pain, burning, bleeding, and dyspareunia [5]. Other symptoms have also been described, such as pruritus and local burning, which may precede the appearance of a palpable mass [6]. This type of tumor occurs in advanced stages. Therefore, local recurrence rates are high, with an unfavorable prognosis compared to other vulvar cancers [7], as they often metastasize to nearby nerves. In this context, there are no specific guidelines on treatment recommendations; given the small number of reported cases, they are generally treated as vulvar cancer [8], with complete surgical removal being the mainstay of treatment. Simple excision as well as simple and radical vulvectomy with or without lymphadenectomy are the most common types of surgery [8]. Radiotherapy and chemotherapy are also often recommended as adjuvant treatment [9,10].

This case study describes the clinical presentation, diagnosis, and treatment used after histopathological confirmation of adenoid cystic carcinoma of the Bartholin's gland, bearing in mind that management is currently still controversial. In our case, right hemivulvectomy with inguinal lymphadenectomy was proposed, with the edges of the lesion free of neoplasia in the frozen section report.

## Case presentation

A 30-year-old mestizo patient with a personal medical history of Barrett's esophagus, under clinical follow-up and treatment, attended a consultation in 2025 at an oncology hospital in Guayaquil, Ecuador, due to a lesion in the right vulvar region that had been causing moderate pain and itching for several months. The pain was initially intermittent until it became constant and limiting. She denied hematochezia, irregular vaginal bleeding, and urinary problems. Physical examination revealed a hard, fixed tumor on the labia majora, sensitive to palpation and attached to the surrounding structures, approximately 2 cm in diameter, without rectal infiltration. Initial laboratory tests and complementary imaging studies were requested, including a complete blood count, blood chemistry, electrolytes, hormone tests, and other special laboratory studies, all of which were within acceptable parameters. Additional imaging tests were also requested: ultrasound examination confirmed a tumorous lesion at the level of the labia majora and perineum.

Given the suspicion of an unknown pathological process in the vulva, an incisional biopsy of the vulvar region was scheduled a few days later. The procedure was performed under general anesthesia, under strict aseptic and antiseptic conditions. The fragments obtained showed adenoid cystic carcinoma (ACC) of the bartholin gland.

**Figure 1:**
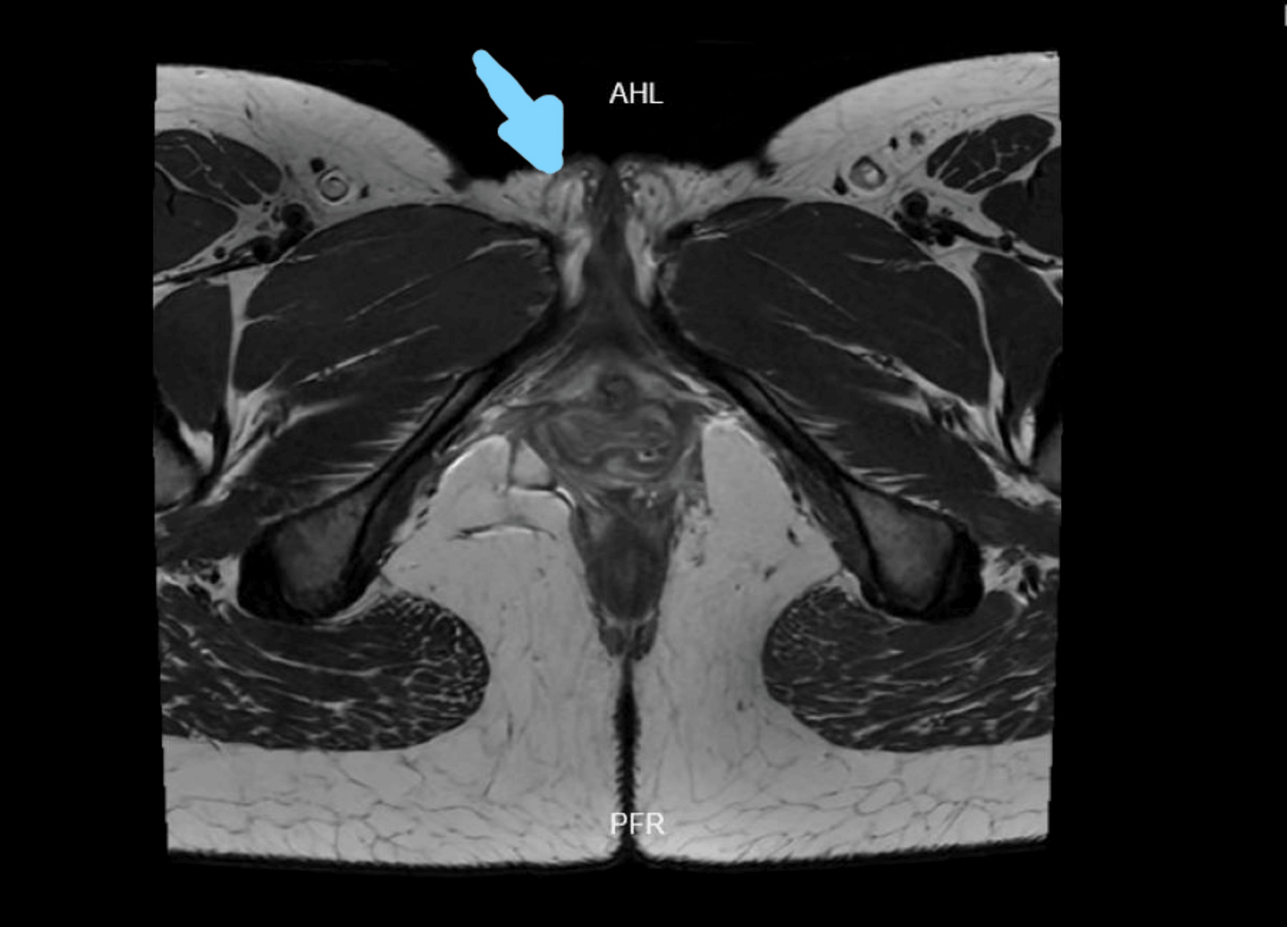
Nuclear magnetic resonance imaging of the pelvis without contrast enhancement (T2) The image shows a tumor lesion, with iso-, hypo-, and hyperintense areas measuring 2.3 cm in diameter, at the perineal level toward the perineal body topography, extending toward the right labia majora (blue arrow).

An RMN (Risonanza Magnetica Nucleare, which means magnetic resonance imaging) of the pelvis was also requested, which reported iso-, hypo-, and hyperintense areas with a diameter of 2.3 cm, at the perineal level toward the perineal body topography, extending toward the right labia majora. No alterations in signal intensity were observed toward the left labia majora and clitoris. No alterations were observed at the level of the sphincter complex or toward the anterior triangle of the perineum. The right and left inguinal lymph nodes retained their shape and hilium, with slight thickening of their cortex (Figure 1).

**Figure 2:**
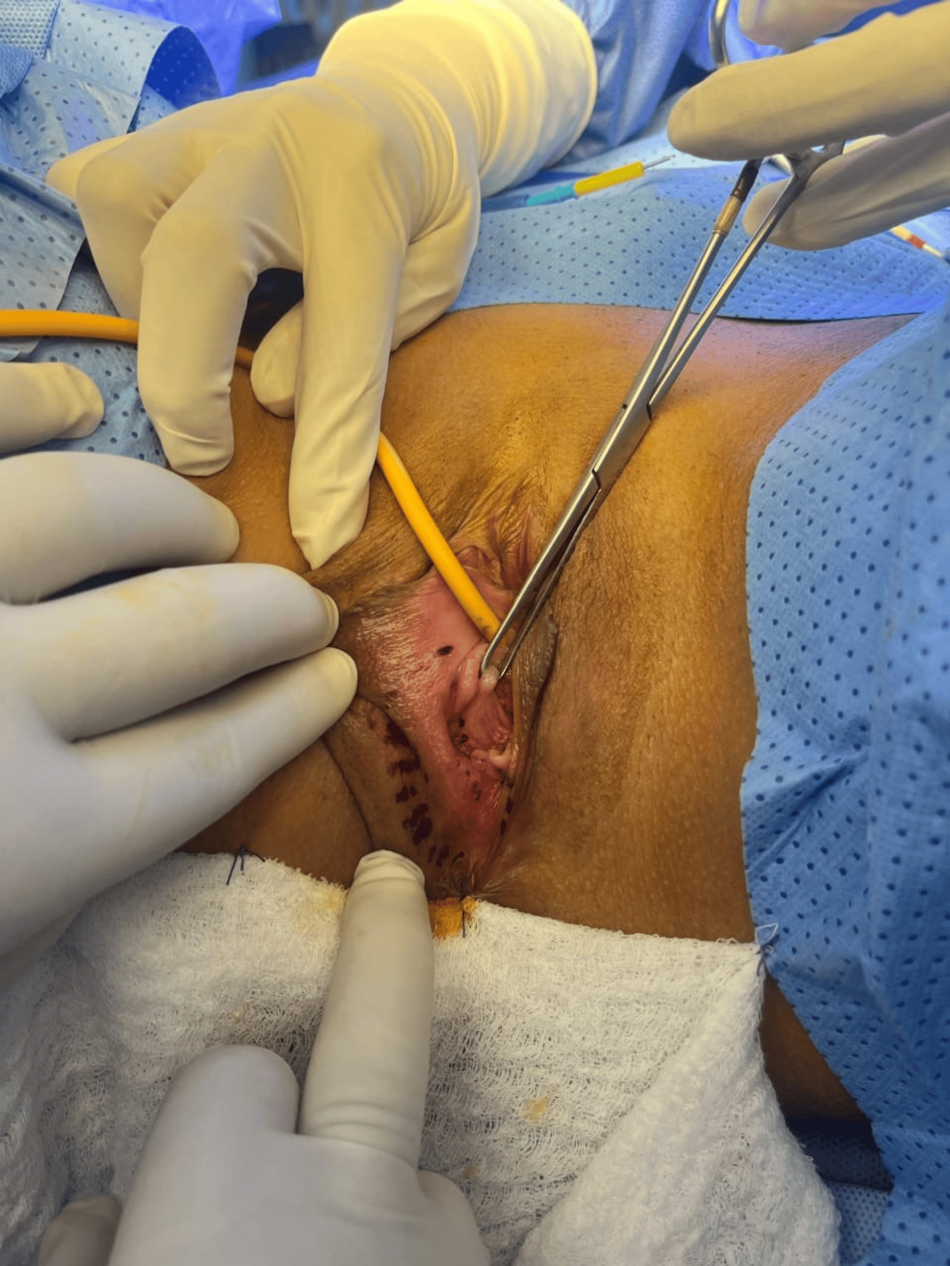
Intraoperative finding The image shows a tumor measuring approximately 2 x 1.5 cm in diameter involving the right labia majora and minora, vaginal introitus mucosa, and perianal region.

**Figure 3:**
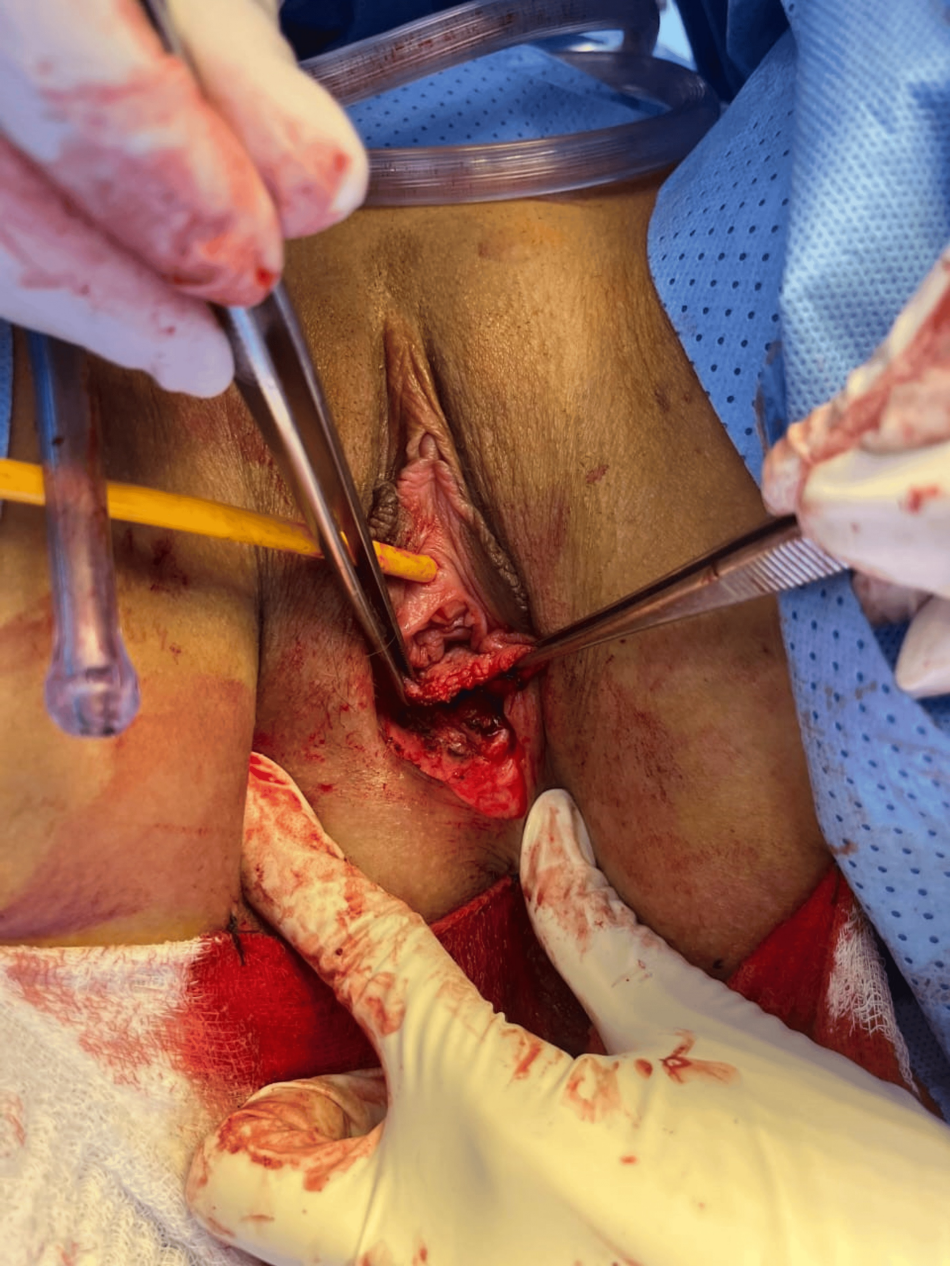
Intraoperative image The intraoperative image shows the procedure: right hemivulvectomy + excision of tumor lesion.

The gynecologic oncology service, in conjunction with the plastic surgery department, performed the following surgical procedures: right hemivulvectomy with plastic reconstruction, sentinel lymph node biopsy, cryotherapy, and right inguinal lymphadenectomy. Intraoperative findings revealed a tumor measuring approximately 2 × 1.5 cm involving the right labia majora and minora, extending from the vaginal introitus mucosa to the perianal region. A lymph node conglomerate was identified in the right inguinal region, with the largest node measuring 1.5 cm. Frostbite report showed edges of right labia lesion free of neoplasia, and the right inguinal region lymph node was negative for malignancy (Figures 2, 3).

**Figure 4:**
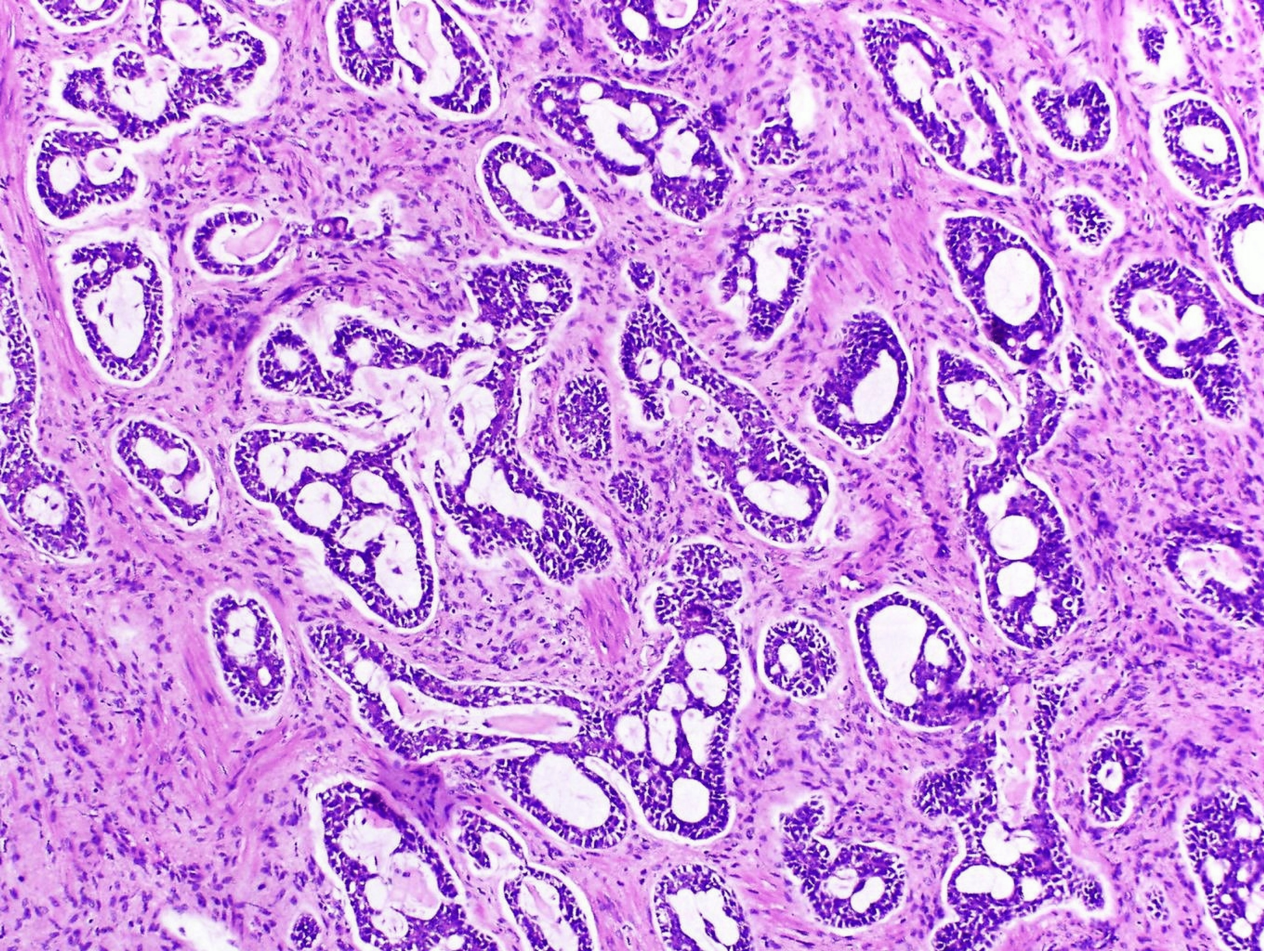
Microscopic images Epithelial proliferation in the form of basaloid nests with microcystic architecture infiltrating the fibrous stroma, intermingling with glandular acini characteristic of the Bartholin's gland, was observed.

Immunohistochemical tests were also performed, with the following results: smooth muscle actin (+), CD117 (+), P63 (+), cytokeratin 7 (+), S100 (+), and KI67 (+) of 20%. The histopathological and immunohistochemical findings were an ACC originating in the Bartholin's gland (Figure 4).

The procedure was completed without complications, with minimal bleeding. The patient was discharged three days after surgery, tolerating food and passing stool.

In subsequent checkups, no masses were palpable on physical examination, and the surgical wound was healing well. Finally, four months after surgery, the patient remains recurrence-free and continues to undergo periodic checkups by the gynecological surgery and oncology departments.

## Discussion

Bartholin's gland carcinoma is a rare neoplasm, accounting for approximately 0.1% to 5% of all malignant neoplasms of the vulva [1,2]. It occurs most frequently in postmenopausal women, although isolated cases have also been reported in women aged 29 years [2], as in the case of the patient mentioned in this study. Malignant neoplasms of the Bartholin's gland include several histological subtypes, such as adenocarcinoma, squamous cell carcinoma, cystic transitional cell carcinoma, and undifferentiated adenocarcinoma. Adenocarcinoma and squamous cell carcinoma are the most common malignant neoplasms, while ACC is considered a very rare malignant genital pathology among gynecological tumors [3,4]. ACC can occur at various sites, including the nasopharynx, breast, lung, major and minor salivary glands, and other sites containing secretory glands, but vulvar localization is extremely rare [5]. Current evidence suggests that the etiology is not related to human papillomavirus (HPV), as in other types of malignant vulvar pathologies; therefore, the mechanisms remain unknown. However, recent studies have shown that chromosomal translocation affecting the genes encoding the MYB and NFIB transcription factors is involved in the development of adenocarcinoma, regardless of its anatomical location of origin [6]. Pregnancy has also been proposed as an independent risk factor [6,8].

Bartholin's gland carcinoma does not present distinctive clinical manifestations, making it difficult to suspect or diagnose in clinical practice [7]. Diagnosis is delayed in up to 50% of cases due to the lack of symptoms, as well as frequent misdiagnosis as a Bartholin's cyst, abscess, or even endometriosis. In this particular case, the patient was young, with no associated risk factors or previous history related to the pathology, and the symptoms were nonspecific. However, when Bartholin’s gland carcinoma (BGC) is suspected, patients often undergo multimodal preoperative imaging, with T2-weighted magnetic resonance imaging being the most appropriate modality for identifying the primary tumor and accurately determining its dimensions [2,8]. The differential diagnosis includes ACC and squamous cell carcinoma (SCC). This differential diagnosis is based primarily on morphology; ACC lacks intraluminal hyaline material, and tumor cells have fewer pleomorphic nuclei and show less mitotic activity [9].

To diagnose this type of tumor, certain characteristics must be present, such as involvement of the anatomical region of the Bartholin's gland, histology compatible with its origin, and absence of a concomitant primary tumor in other areas [10]. These characteristics were met in the case of our patient, as the preoperative findings, complementary images, and characteristics of the pathology report allowed us to confirm the diagnosis of Bartholin's gland carcinoma. Considering that most reported cases are misdiagnosed and poorly managed due to the lack of scientific evidence on the subject, even with early and correct diagnosis and successful local treatment, most patients die of metastasis within 10 years [11], as this pathology presents a mechanism known as perineural invasion, which indicates a poor prognosis with a higher probability of local recurrence [11,12].

There is no consensus on the treatment of this pathology, so it must be individualized for each patient [12,13]; surgical resection is the treatment of choice. The surgical procedure can be performed by wide local excision, hemivulvectomy, simple vulvectomy, and radical vulvectomy with or without inguinal or femoral lymphadenectomy [13]. However, lymphadenectomy is not recommended unless there is clinical or radiological suspicion of lymph node dissection, as this tumor usually metastasizes to distant organs before affecting locoregional lymph nodes [13]. Sentinel node biopsy is recommended for tumors < 4 cm or > T1a without suspicious nodes on examination/imaging; this procedure is not feasible when the tumor involves the vagina, anus, or urethra [11,14].

In this context, in our patient, the tumor was located on the right labia majora, so surgical resection was performed as a therapeutic option, preferring right hemivulvectomy plus inguinal lymphadenectomy with the edges of the lesion free of neoplasia in the frozen section report. Regarding inguinal lymphadenectomy, although there is controversy regarding this complementary procedure, especially in our patient who did not have lymph node involvement, it was performed considering the possibility of metastasis in the inguinal-femoral lymph nodes [3,14]; if this procedure is performed, it should be limited to at least the ipsilateral side of the tumor [14,15]. In addition to primary treatment, adjuvant radiotherapy or chemotherapy has been suggested for patients with deep local infiltration, positive surgical margins, or recurrence, as these can reduce local recurrence rates, especially when positive surgical margins are identified [14]. Chemotherapeutic agents, including cisplatin, are commonly used in combination with radiotherapy for the dual benefit of enhancing its effects by inducing radiosensitization and, at the same time, inducing direct cytotoxicity [15].

As for oncological margins, the goal of resection is to obtain tumor-free pathological margins. A tumor-free surgical margin of at least 1 cm reduces local recurrence. However, narrower margins are acceptable when the tumor is located near midline structures, such as the clitoris, urethra, or anus, and preservation of these structures is desired [11,15].

An important prognostic factor is the presence of the KI-67 marker, which according to the literature, when elevated above 30%, is associated with greater aggressiveness and tumor spread [15].

ACC is associated with high recurrence rates and has an overall survival rate of 77% at five years, 60% at 10 years, and 45% at 15 years [16]. Regular monitoring is recommended for patients diagnosed for a minimum of five years [17]. However, due to postoperative and post-radiotherapy changes, clinical examination may be suboptimal; therefore, this procedure should be considered at regular intervals [2,17]. The survival time for patients with a positive margin is less than seven years, and the survival time for patients with a negative margin is approximately 15 to 30 years [17].

## Conclusions

ACC of the Bartholin's gland is an extremely rare condition with a low global incidence and an etiology that has not yet been defined. Early and timely diagnosis, management, and treatment can prevent serious complications and reduce recurrence rates. In this particular case, although there has been a favorable clinical outcome following specific surgical treatment (complete surgical resection with clear surgical margins and KI-67 of 20%), with no current clinical or imaging evidence of tumor recurrence, it is necessary to maintain more extensive multidisciplinary follow-up and close monitoring for at least five years, as recommended by current scientific evidence.
